# Gut microbiota-based vaccination engages innate immunity to improve blood glucose control in obese mice

**DOI:** 10.1016/j.molmet.2021.101404

**Published:** 2021-11-25

**Authors:** Brittany M. Duggan, Akhilesh K. Tamrakar, Nicole G. Barra, Fernando F. Anhê, Gabriella Paniccia, Jessica G. Wallace, Hannah D. Stacey, Michael G. Surette, Matthew S. Miller, Deborah M. Sloboda, Jonathan D. Schertzer

**Affiliations:** 1 Department of Biochemistry and Biomedical Sciences, McMaster University, Hamilton, Canada; 2 Farncombe Family Digestive Health Research Institute, McMaster University, Hamilton, Canada; 3 Centre for Metabolism, Obesity and Diabetes Research, McMaster University, Hamilton, Canada; 4 Division of Biochemistry, CSIR-Central Drug Research Institute, Lucknow, 226031, India; 5 Michael G. DeGroote Institute for Infectious Disease Research, McMaster University, Hamilton, Canada; 6 McMaster Immunology Research Centre, McMaster University, Hamilton, Canada; 7 Department of Obstetrics and Gynecology, McMaster University, Hamilton, Canada; 8 Department of Pediatrics, McMaster University, Hamilton, Canada; 9 Department of Medicine, McMaster University, Hamilton, Canada

**Keywords:** Obesity, Diabetes, Insulin resistance, Immunometabolism, Microbiota, T2D, Type 2 Diabetes, FMT, fecal microbial transfer, LPS, lipopolysaccharide, meso-DAP, meso-diaminopimelic acid, MDP, muramyl dipeptide, CD, control diet, HFD, high fat diet, GTT, glucose tolerance test, ITT, insulin tolerance test, OGSIS, oral glucose-stimulated insulin secretion, PGN, peptidoglycan, PRR, pattern recognition receptor, IgG, immunoglobin-G, RED, reciprocal endpoint dilution

## Abstract

**Objective:**

Obesity and diabetes increase circulating levels of microbial components derived from the gut microbiota. Individual bacterial factors (i.e., postbiotics) can have opposing effects on blood glucose.

**Methods:**

We tested the net effect of gut bacterial extracts on blood glucose in mice using a microbiota-based vaccination strategy.

**Results:**

Male and female mice had improved glucose and insulin tolerance five weeks after a single subcutaneous injection of a specific dose of a bacterial extract obtained from the luminal contents of the upper small intestine (SI), lower SI, or cecum. Injection of mice with intestinal extracts from germ-free mice revealed that bacteria were required for a microbiota-based vaccination to improve blood glucose control. Vaccination of *Nod1*^*−/−*^, *Nod2*^*−/−*^, and *Ripk2*^*−/−*^ mice showed that each of these innate immune proteins was required for bacterial extract injection to improve blood glucose control. A microbiota-based vaccination promoted an immunoglobulin-G (IgG) response directed against bacterial extract antigens, where subcutaneous injection of mice with the luminal contents of the lower SI elicited a bacterial extract-specific IgG response that is compartmentalized to the lower SI of vaccinated mice. A microbiota-based vaccination was associated with an altered microbiota composition in the lower SI and colon of mice. Lean mice only required a single injection of small intestinal-derived bacterial extract, but high fat diet (HFD)-fed, obese mice required prime-boost bacterial extract injections for improvements in blood glucose control.

**Conclusions:**

Subversion of the gut barrier by vaccination with a microbiota-based extract engages innate immunity to promote long-lasting improvements in blood glucose control in a dose-dependent manner.

## Highlights

• Subcutaneous injection of gut bacterial extracts improved blood glucose control in mice.
• Microbiota-based vaccination engaged NOD1-NOD2-RIPK2 to alter blood glucose.
• Microbiota-based vaccination promoted a proximal gut IgG response.
• Microbiota-based vaccination altered the composition of the gut microbiome.
• Obese mice required prime-boost injections to improve blood glucose control.

## Graphical abstract

**Figure:**
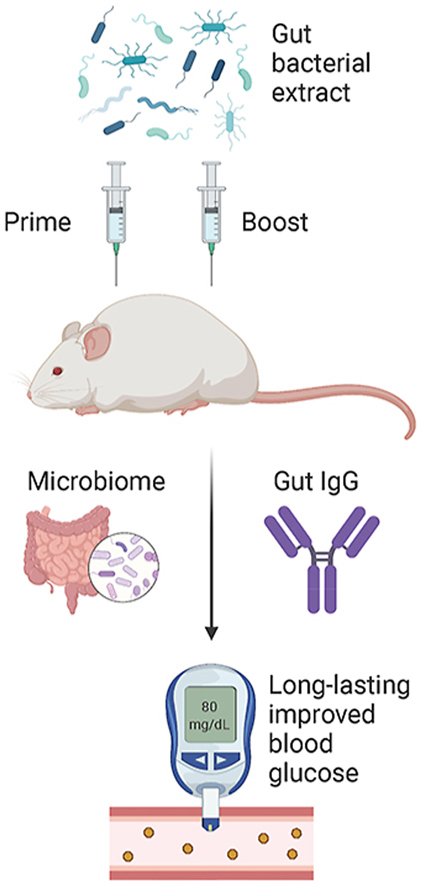

## 1 Introduction

The gut microbiota shapes host innate and adaptive immune responses, which reciprocally regulate the composition and function of the gut microbiota [[1], [2], [3]]. This is relevant to metabolic disease because metabolic inflammation (i.e., metaflammation) contributes to insulin resistance and impaired blood glucose control [4]. A common hypothesis is that obesity is associated with systemic inflammation. Obesity can coincide with low-level inflammation in metabolic tissues that regulate blood glucose, such as adipose and liver [[5], [6], [7]]. Some compartmentalized immune responses in the proximal gut are lower in mouse models of obesity or prediabetes compared to their lean, normoglycemic counterparts [8,9]. For example, there are fewer intestinal immunoglobulin A (IgA)-producing cells and less secretory IgA-associated immune mediators in high fat diet (HFD)-fed obese mice [10]. There are also reductions in Cd4+ and Cd8+ T cells and impaired Th17 immune responses in the ileum of mice fed obesogenic and diabetogenic diets [9,11,12]. These data show that immunological changes during obesity are not restricted to chronically increased inflammation; in some circumstances, obesity is associated with a dampening of the immune responses that may be compartmentalized to specific tissues or cell populations.

The triggers of compartmentalized inflammation during obesity are largely unknown, although it has been proposed that changes in the gut microbiota (i.e., dysbiosis) promote metaflammation and consequently poor blood glucose control during obesity. Obesity is associated with changes in bacterial diversity and composition in rodents and humans and colonizing germ-free mice with obese microbiota promotes adiposity and poor blood glucose control [[13], [14], [15]]. These data suggest a causal role for gut microbiota-derived factors in metabolic and endocrine control [14,[16], [17], [18]]. Consequently, altering the gut microbiota composition and fecal microbial transfer (FMT) have been proposed to mitigate insulin resistance and dysglycemia [[19], [20], [21], [22]]. Which microbial strains are responsible for improved metabolic control is unclear, however specific commensal bacteria have been suggested to protect against aspects of metabolic disease; for example, intestinal abundance of *Bifidobacterium* species and *Akkermansia muciniphila* has been inversely associated with obesity, insulin resistance and type 2 diabetes (T2D) [[23], [24], [25], [26], [27], [28]]. It is still not clear how the multiple functions of gut microbe communities or specific bacterial strains can be harnessed to alter blood glucose, but emerging evidence shows that gut microbes impact host health through changes in circulating metabolites [29].

Bacteria and their components or metabolites can subvert the intestinal barrier and reach extra-intestinal tissues to exert compartmentalized immune and metabolic responses [30,31]. Increased systemic bacterial load is proposed to worsen blood glucose control, where T2D alters bacterial DNA signatures and indicators of live bacteria in adipose tissue of humans with obesity [30,31]. Live bacteria are not required to alter host immunity and metabolism. Bacterial-derived factors, cell components and metabolites, collectively termed postbiotics, can alter host blood glucose homeostasis [32]. For example, subverting the gut barrier by injection or infusion with specific bacterial cell wall components such certain types of lipopolysaccharide (LPS) and meso-diaminopimelic acid (meso-DAP)-containing muropeptides, triggers inflammatory responses and exacerbates insulin resistance and dysglycemia in mice [[33], [34], [35]]. Conversely, infusion of LPS with underacylated lipid A or repeated injection of the bacterial cell wall component muramyl dipeptide (MDP) lowers adipose tissue inflammation and mitigates insulin resistance in obese mice by engaging a Nucleotide Binding Oligomerization Domain Containing 2 (NOD2) innate immune response [[36], [37], [38]]. Single bacteria factors can have opposing effects on blood glucose, but the net effect of various systemic postbiotics derived from the gut microbiota is not yet clear.

Bacteria can alter immune responses beyond acute pro-inflammatory or anti-inflammatory actions. Immunological memory allows for a rapid and effective response upon secondary encounter with the same antigen through adaptive immunity [39]. Innate immune cell memory responses are emerging, but poorly defined in metabolic disease [[40], [41], [42]]. A seminal study showed that subcutaneous injection of a crude total extract derived from luminal contents from the most distal 10 cm segment of the small intestine (SI) (referred to by authors as the ileum) of mice, generated an adaptive immune response that partially protected mice from developing glucose intolerance and insulin resistance [43]. This bacterial “vaccination-like” strategy provided long-lasting improvements in blood glucose control when used prior to feeding mice a diabetogenic diet. Improved glycemia required an adaptive immune system, since *Rag1*^*−/−*^ mice lacking lymphocytes were refractory to bacterial vaccination-induced effects on glucose tolerance, and adoptive transfer of immune cells from bacterial-vaccinated mice into naïve mice protected against dysglycemia induced by a diabetogenic diet in mice [43]. This previous study used a diabetogenic diet that did not induce obesity and the role of the innate immune system was not investigated. Here, we tested the net effect of postbiotics found in the gut microbiota on immune responses and blood glucose regulation in lean and diet-induced obese mice, using a microbiota-based vaccination strategy. We found that subcutaneous injection of bacterial luminal extracts derived from the lower SI elicited an IgG response in the lower SI, altered the composition on the gut microbiome, and promoted long-lasting improvements in blood glucose control. Microbiota-based vaccination with extracts from the upper SI and the cecum also improved blood glucose control. We found that a microbiota-based vaccination strategy required a NOD2-mediated innate immune response, and obese mice required prime-boost injections to improve glucose control.

## 2 Methods

### 2.1 Mice & materials

All animal procedures for this study were approved by the McMaster University Animal Research Ethics Board in accordance with the guidelines of the Canadian Council of Animal Care. All mice were a minimum of 8 weeks old before experiment initiation. Mice were maintained on a 12-h light/dark cycle, and experiments were performed on multiple cohorts of mice born from different parents at different times of the year. WT (C57BL/6J), *Nod1*^*−/−*^, *Nod2*^*−/−*^ and *Ripk2*^*−/−*^ mice used for experiments were bred in-house under specific pathogen-free conditions at McMaster University. Germ-free mice were obtained from the Farncombe Gnotobiotic Unit of McMaster University. All animals were fed a control diet (CD) (17% kcal from fat, 29% kcal from protein, 54% kcal from carbohydrate: cat# 8640 Teklad 22/5, Envigo). Where indicated, mice were fed an obesity-promoting 60% HFD (60% kcal from fat, 20% kcal from protein, 20% kcal from carbohydrate: cat# D12492). Glucose tolerance tests (GTTs) and insulin tolerance tests (ITTs) were performed in 6-h-fasted, conscious mice. Oral glucose-stimulated insulin secretion tests (OGSIS) were performed in 12 h-fasted, conscious mice [44]. The dose of d-glucose (Sigma–Aldrich) or insulin (NovoRapid, Novo Nordisk) and route of administration using intraperitoneal (i.p.) injection or oral gavage (p.o.) is indicated in each figure caption for each experiment. Blood glucose was determined by tail vein blood sampling using a handheld glucometer (Roche Accu-Check Performa). During OGSIS tests, blood samples were collected via tail-vein sampling at time (t) = 0-, 10- and 60-min post-glucose gavage (4 g/kg). Blood was centrifuged for 10 min at 4 °C and 10,000 *g*, and plasma fraction was collected and stored at −80 °C. Plasma insulin levels were assessed using high sensitivity mouse insulin ELISA kit (Toronto Bioscience, Cat# 32270) and measured with a Synergy H4 Hybrid reader (Biotek Instruments). Area under the curve (AUC) of blood glucose or blood insulin vs. time was calculated for GTT, ITT, and OGSIS using GraphPad Prism 6 software (with baseline Y values set to 0).

### 2.2 Collection and preparation of intestinal extracts for vaccination

**Supplemental Figure 1:**
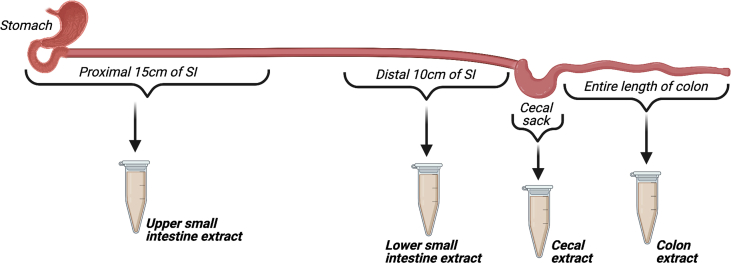
**Defined length of intestinal segments used for bacterial extracts.** Luminal contents were extracted from the proximal 15 cm of small intestine (SI) and defined as the upper SI bacterial extract. Luminal contents of the distal 10 cm of distal SI were collected and defined as the lower SI extract. Luminal contents of the entirety of cecal sack were used for Cecal extracts, and luminal contents of the entire length of colon were used for colon extracts.

Each intestinal extract contained the luminal contents of 3 donor mice of the same sex as the recipient mice. Groups of 3 WT/J donor mice, aged 10–14 weeks, were used for preparation of intestinal extracts, and mice were fed a control diet (CD) or high fat diet (HFD) for 4 weeks prior to euthanization via cervical dislocation. The entire length of the both the small and large intestine were removed with sterile tools. Luminal contents of each intestinal section were collected via gentle and thorough pressurization into a clean tube containing 500 μL DPBS on ice, using separate tools for each section. The upper SI was defined as the proximal 15 cm of SI, measured distally from the pyloric sphincter. The lower SI was defined as the distal 10 cm of SI, starting from cecum and measuring proximally. The entire contents of the cecal sack were collected for cecum extracts. The entire length of the colon from the cecum to the rectum was collected for colon extracts. The defined lengths of intestinal segments used are depicted in Supplemental Figure 1. Upon collection, all luminal contents were vigorously vortexed for 1 min to ensure thorough mixing. For fecal extracts, a single fecal pellet was collected from each of the 3 donor mice prior to euthanization and mechanically homogenized at 4.5 m/s for 1 min using a FastPrep-24 tissue homogenizer (MP Biomedicals) and two plastic beads. Germ-free intestinal extracts were prepared from 3 age-matched germ-free mice immediately upon export from the Farncombe Gnotobiotic Unit at McMaster University. Euthanization and intestinal extracts were prepared in a level II biosafety hood to limit contamination with ambient microbes. All extracts were centrifuged (4 °C, 7500 rpm, 5 min) to pellet debris and supernatant was transferred into fresh tubes. Supernatant was sonicated for 1 min (Fisher Scientific 20kHx sonicator), aliquoted, and stored at −80° until use. Extracts were stored for a maximum of 4 months, and multiple separate collections of extracts were tested across multiple cohorts of mice. The pooled intestinal extract from 3 mice were combined and used to inject (i.e., vaccinate) multiple mice of the same sex for each experiment.

### 2.3 Microbiota-based vaccination

Luminal contents from the lower SI were used unless otherwise indicated. Specific dilutions of intestinal extracts were prepared fresh on the day of injection in recipient mice in dPBS (50-20,000x dilution). WT (C57BL/6J, male and female) and *Nod1*^*−/−*^, *Nod2*^*−/−*^ and *Ripk2*^*−/−*^ male mice, aged 10–18 weeks old, received a single subcutaneous injection of intestinal extract (200 μL, *s.c.*, of upper SI, lower SI, cecal, colon or fecal extract, as indicated). Glucose tolerance, insulin tolerance, or oral glucose-stimulated insulin secretion was assessed 5 weeks after injection, as indicated.

For the “prime-boost” vaccination model, male mice received a first injection of lower SI extract (200 μL, *s.c.*) after 8 weeks of HFD-feeding, and a second injection was administered after 12 weeks of HFD-feeding. Glucose tolerance was assessed after one injection at 12 weeks and two injections at 16 weeks of HFD-feeding. In the CD-to-HFD prime-boost model, male mice were fed a CD, injected once, and glucose tolerance was assessed at 5 weeks of CD feeding. 5 weeks after the initial injection, CD mice were switched to a 60% HFD. After 8 weeks on a HFD mice were injected a second time. Glucose tolerance was tested 4 weeks after the secondary ‘boost’ injection, after a total of 12 weeks of HFD-feeding.

### 2.4 Quantification of IgG

Serum, ileum, cecal, and colon samples were harvested from vaccinated male mice 6 weeks after the vaccination event. Whole blood was collected via facial vein sampling in conscious mice. Mice were immediately euthanized by cervical dislocation, and intestinal segments were immediately excised. Blood was clotted for 20 min and centrifuged for 10 min at 4 °C and 10,000×*g*. The serum fraction was collected and stored at −80° until assay. Intestinal samples (ileum, cecum, colon) were homogenized in 1.5 mL Rino Tubes (Next Advance, Troy, NY, USA) containing 1.6 mm stainless steel beads (Next Advance, Troy, NY, USA) and 500 μL dPBS using the Bullet Blender Gold (Next Advance) at maximum speed for 10 min at 4 °C, and then centrifuged for 5 min at 8,000 g. Supernatants were collected and stored at −80° until assayed. Protein concentration of lower SI extract was determined by Pierce BCA Protein assay (Thermo Scientific, Waltham, MA, USA) according to manufacturer's instructions and 96-well NUNC- Maxisorp plates (Thermo Scientific, Waltham, MA, USA) were coated overnight at 4 °C with 2 μg/mL of the same lower SI extract used for vaccination. The extract was diluted in bicarbonate-carbonate coating buffer (pH 9.4). The next day antigen-coating buffer was discarded and plates were blocked by shaking for 1 h at 37 °C with reagent diluent (0.5% bovine serum albumin [BSA], 0.02% sodium azide, in 1X Tris-Tween wash buffer −10X composed of 0.024% Tris, 0.0876% Sodium Chloride, 0.00373% Potassium Chloride, 0.005% Tween-20 in 800 mL MilliQ, adjusted to pH 7.4 with 3M HCl, final volume 1000 mL). After blocking, samples were serially diluted from a 1:10 (serum) or 1:2 starting dilution (ileum, cecum, colon). Samples were incubated for 1 h, shaking at 37 °C. Following sample incubation, plates were washed three times with 1X Tris-Tween wash buffer. A goat anti-mouse IgG-biotin antibody (Southern Biotech) was diluted 1:5000 in reagent diluent and added to all wells. Plates were again incubated for 1 h at 37 °C with shaking, followed by three washes with 1X Tris-Tween buffer. A streptavidin-alkaline phosphatase secondary antibody (Southern Biotech) was diluted 1:2000 in reagent diluent and added to all wells followed by another 1 h incubation at 37 °C with shaking. Following this incubation, 3 more washes with 1X Tris-Tween buffer were performed, the final wash was removed, and pNPP one component microwell substrate solution (Southern Biotech) was added to each well. After 10 min of developing, the reaction was quenched with 3N sodium hydroxide. The optical density (O.D.) at 410 nm was read on a Spectramax I3 (Molecular Devices, San Jose, CA, USA). IgG endpoint titers were defined by the lowest dilution at which the O.D. was three standard deviations above the mean of the blank wells.

### 2.5 Bacterial profiling

Isolation of DNA from ileal and colon contents was done using mechanical and enzymatic lysis [45]. The V3-4 region of the 16S rRNA gene was PCR amplified with barcode tags compatible with Illumina technologies, and the Illumina MiSeq platform was used to sequence amplified DNA products. Details are available at www.surettelab.ca/protocols. A custom pipeline was used to process the FASTQ files. Dada25 [46] was used to assign reads to Amplicon Sequence Variants (ASVs) and assign taxonomy with the Ribosomal Database Project (RDP) Bayesian classifier using the Silva 132 database [47]. ASV assignments were converted to relative abundance before β-diversity calculations to account for depth of coverage and to normalize across samples. QIIME and R scripts were used to calculate β-diversity and to perform statistical tests.

### 2.6 Bacterial 16S quantification and bacterial viability

Sonicated, undiluted intestinal extract (300 μL) was processed using ZymoBIOMICS DNA kit (cat# D4300, Zymo Research Corporation), according to manufacturer's instructions, with the addition of 2 enzymatic lysis steps following mechanical homogenization. These additions to the protocol consisted of an incubation with lysis solution 1 (50 mg/mL lysozyme and 20% RNase), rocked at 37 °C for 1 h, and incubation with lysis solution 2 (25 μL of 25% SDS, 25 μL of 5M NaCl, 50 μL of 10 mg/mL Proteinase K) at 60 °C for 30 min qPCR for bacterial 16S rRNA gene was then conducted, as described [48]. In brief, master mix comprised 50% BioRad SsoFast EvaGreen supermix (cat#172–5200), 0.5% forward primer 926f∗ (AAACTCAAKGAATTGACGG), 0.5% reverse primer 1062r (CTCACRRCACGAGCTGAC), 5% 10 mg/mL BSA, 39% ultrapure H_2_0, and 1% standard or unknown sample (purified intestinal extract). Purified genomic DNA from *Escherichia coli* were used to generate a standard curve between 10 and 100 ng/μL. The qPCR was performed using a Bio-Rad CFX96 thermocycler and all calculations were performed on the Bio-Rad CFX96 software. Bacterial viability in the extracts was assessed in SI extracts that were diluted 2x in saline or ethanol and incubated 20 min at RT. Inside an anaerobic chamber, 100 μL of SI extracts were pipetted into Brain Heart Infusion (BHI) agar containing plates. BHI was supplemented with 0.5 g/L l-cysteine hydrochloride hydrate, 10 mg/L hemin, and 1 mg/L Vitamin K or rumen fluid. All plates were incubated anaerobically for 5 days. Identification of colonies was performed using MALDI-TOF mass spectrometry (Microflex LRF, Bruker).

### 2.7 Statistical analysis

Individual data points indicate separate mice, and data is expressed as mean ± standard error of the mean (SEM). Comparisons were made using unpaired, two-tailed Student's t-test where two variables are compared. ANOVA was used for comparison of more than 2 variables and Tukey's post-hoc test was used when appropriate. Analysis of microbial populations was conducted in R. Partitioning of the variance in the microbiota was done with a Permutational multivariate analysis of variance (PERMANOVA) on Bray–Curtis dissimilarities calculated from relative amplicon sequence variant (ASV) abundance. The Wilcoxon rank sum test was used for pairwise comparisons. Adjustment for the false discovery rate (FDR) was calculated with the Benjamini-Hochberg method. R packages used for data analysis and visualization included vegan, ggplot2, tidyr, dplyr, ggtree, and corrplot. Significance was accepted at *p* < 0.05. Graphpad Prism 6–9 software was used for all analysis and diagrams were created using BioRender.com software. All data and R scripts generated in this study are available upon reasonable request.

## 3 Results

### 3.1 Microbiota-based vaccination with proximal-gut bacterial components improves blood glucose control in lean male mice

**Figure 1:**
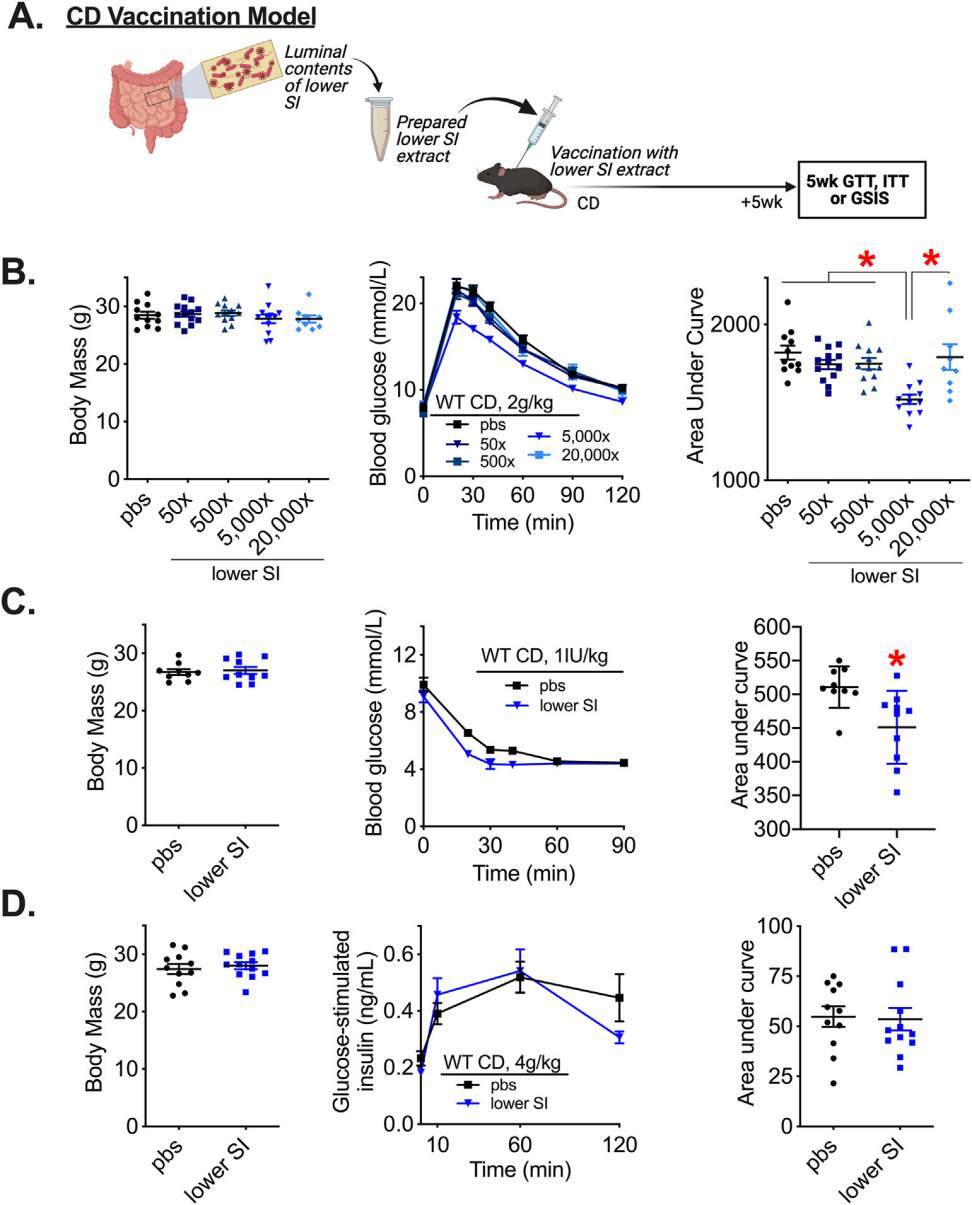
**Subcutaneous injection of a specific concentration of lower small intestinal contents improves glucose and insulin tolerance in lean male mice.** A) Experimental vaccination model of control diet (CD)-fed mice given a single subcutaneous injection with a previously prepared lower small intestinal (SI) extract. Glucose tolerance, insulin tolerance, and glucose-stimulated insulin secretion were assessed 5 weeks after vaccination. B) Body mass, blood glucose vs. time and quantified area under the curve during a glucose tolerance test (GTT) (2 g/kg, i.p.) in CD-fed WT male mice 5 weeks after vaccination with various dilutions (50x-20,000x) of lower SI extract (n = 9–13). C) Body mass, blood glucose vs. time and quantified area under the curve during an ITT (1IU/kg, i.p.) and D) Body mass, plasma insulin concentration vs. time and quantified area under the curve during a GSIS (4 g/kg, p.o.) in CD-fed WT male mice 5 weeks after vaccination with a 5000x dilution of lower SI extract (n = 10–12). ∗Denotes significant difference from control group injected with saline (pbs) determined by t-test or one-way ANOVA, where appropriate (p < 0.05). Each symbol represents a mouse and other values shown are the mean +/− SEM.

The luminal contents from the lower SI were collected, sonicated, and pooled from a group of 3 wild type C57Bl/6J male mice fed a control diet (CD). These lower SI extracts were used for future subcutaneous injection (i.e., vaccination) into recipient male mice. Male mice fed a CD were vaccinated with a single injection of lower SI extract 5 weeks before metabolic testing (Figure 1A). We found that injection of lower SI extracts caused a dose-dependent change in blood glucose during a glucose tolerance test (GTT), but did not alter body mass (Figure 1B). Diluting lower SI extracts by 5000x lowered blood glucose levels during a GTT, compared to less dilute (50x, 500x) and more dilute (20,000x) preparations of the bacterial extract, demonstrating that a critical concentration of lower SI extract was required to alter glycemia. This concentration of intestinal extract required for lowering blood glucose is consistent with published results [43] and was used for all subsequent tests unless otherwise specified. We found that injection of lower SI extracts (5000x dilution) lowered blood glucose levels during an insulin tolerance test (ITT) (Figure 1C) but did not alter insulin secretion during an oral glucose load (Figure 1D).

**Figure 2:**
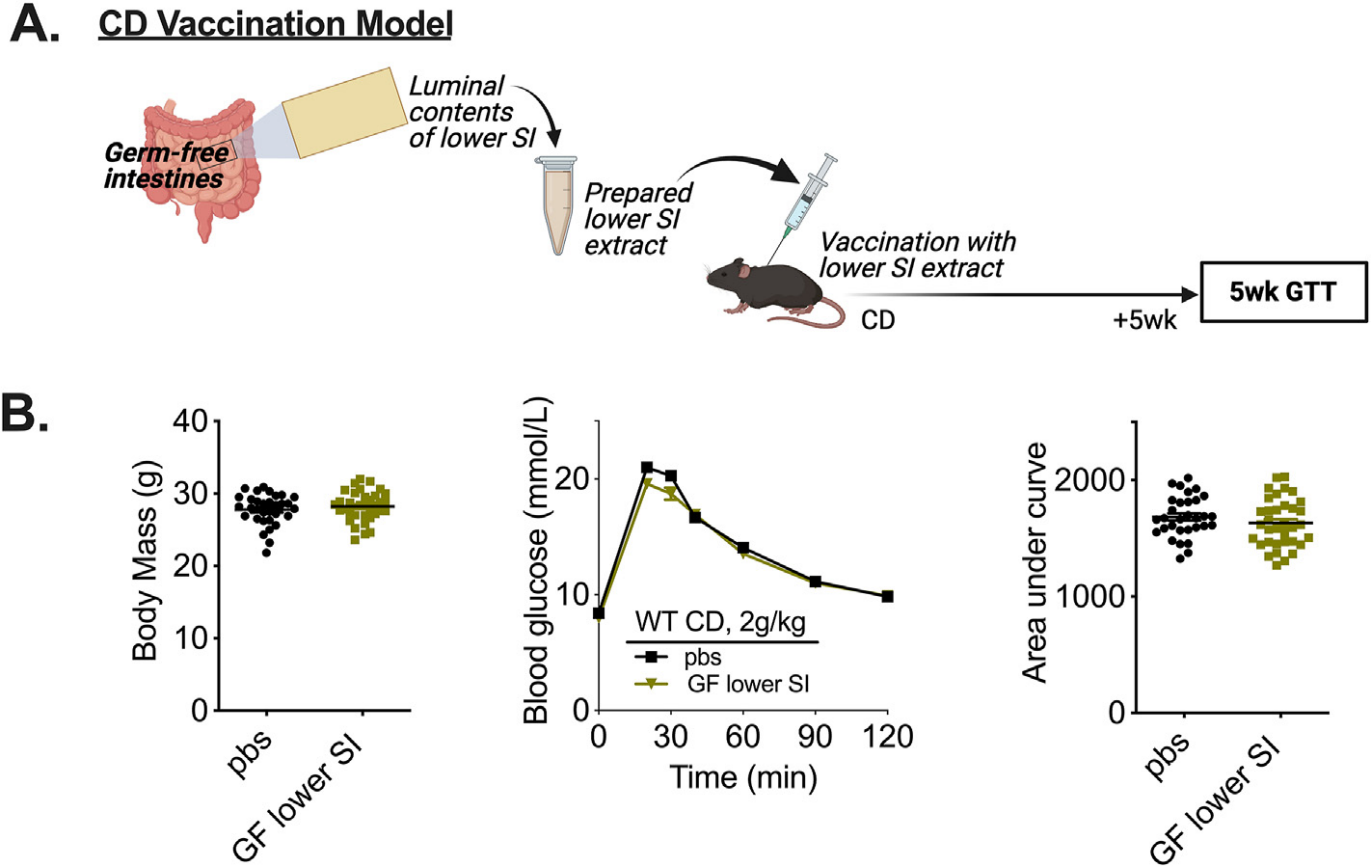
**Bacteria are required for a microbiota-based vaccination to improve blood glucose control in lean male mice**. A) Experimental vaccination model of control diet (CD)-fed mice given a single subcutaneous injection with an extract prepared from the lower small intestinal (SI) luminal contents of germ-free mice. Glucose tolerance was assessed 5 weeks after the vaccination event. B) Body mass, blood glucose vs. time and quantified area under the curve during a glucose tolerance test (GTT) (2 g/kg, i.p.) in CD-fed WT male mice 5 weeks after vaccination with germ-free lower SI extract (n = 31–35). ∗Denotes significant difference from control (pbs) group determined by unpaired t-test (p < 0.05). Each symbol represents a mouse and other values shown are the mean +/− SEM.

**Supplemental Figure 2:**
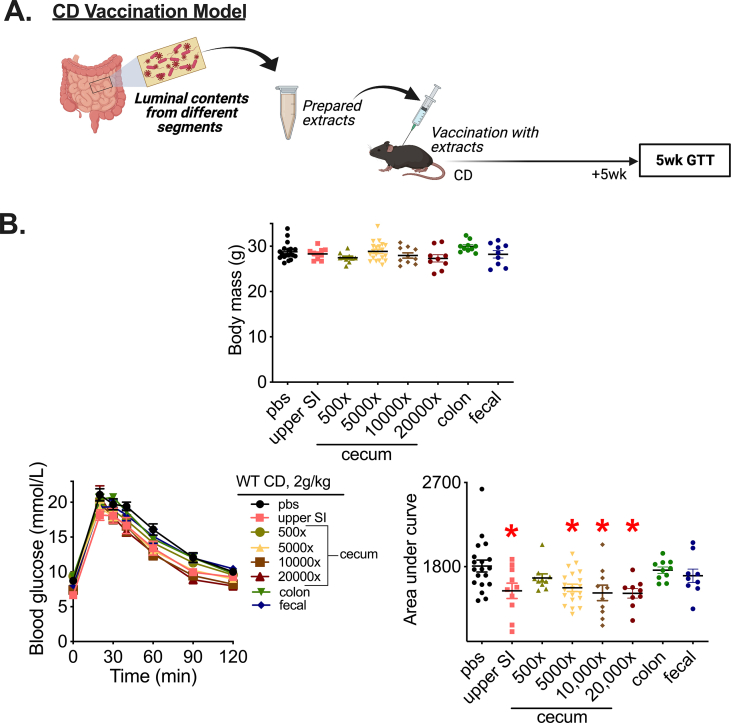
**Microbiota-based vaccination with a specific dose of extracts from different gut segments improves blood glucose control in male mice.** A) Vaccination model of control diet (CD)-fed male mice given a single subcutaneous injection with extracts prepared from different intestinal segments (upper small intestine (SI), cecum, colon) or from a homogenized fecal pellet (‘fecal’) from male mice. Glucose tolerance was assessed 5 weeks after the vaccination event. B) Body mass. Blood glucose vs. time and quantified area under the curve during a glucose tolerance test (GTT) (2 g/kg, i.p.) in CD-fed WT male mice 5 weeks after vaccination (5000x dilution of upper SI, colon and fecal extracts and a range of 500–20,000x dilutions of cecal extracts were tested; n = 9–10). ∗Denotes significant difference from control group injected with saline (pbs) determined by one-way ANOVA (p < 0.05). Each symbol represents a mouse and other values shown are mean +/− SEM.

Previously published results demonstrated that lower SI (i.e., “ileal”) extracts that were prepared from antibiotic-treated or germ-free mice did not alter glycemic control in vaccinated mice [43], suggesting that the antigen(s) responsible for the glucose-lowering effects were of microbial origin. Poor reproducibility of microbial-mediated phenotypes across different research facilities can limit robust conclusions; therefore, we sought to test germ-free (GF) extracts in our microbiota vaccination model to confirm antigen(s) responsible for the glucose-lowering effects of lower SI extracts were derived from bacteria. Lower SI extracts were collected from several cohorts of germ-free (GF) mice and used to vaccinate CD-fed male mice (Figure 2A). Vaccination with lower SI extracts from male GF mice did not alter body mass or blood glucose levels in CD-fed male mice (Figure 2B). The glucose-lowering effects of lower SI extracts are mediated by microbial components present in the intestinal milieu, consistent with previously published results [43]. We tested the microbiota vaccination procedure using extracts harvested from different intestinal segments spanning the length of the small and large intestine in mice (See Supplemental Figure 1 for definitions of distinct segments). In addition to the lower SI, extracts collected from the luminal contents of the upper SI and cecum, when delivered at specific doses, lowered blood glucose levels during a GTT. This occurred independent of changes in body mass (Supplemental Figure 2). Taken together, these data show that small intestinal and cecal gut segments contain microbial factors (i.e., postbiotics) that improve glycemic control in lean male mice after a single vaccine-like subcutaneous injection.

### 3.2 Microbiota-based vaccination with bacterial extracts derived from both lean and obese mice improve glycemic control and effects are independent of sex

**Figure 3:**
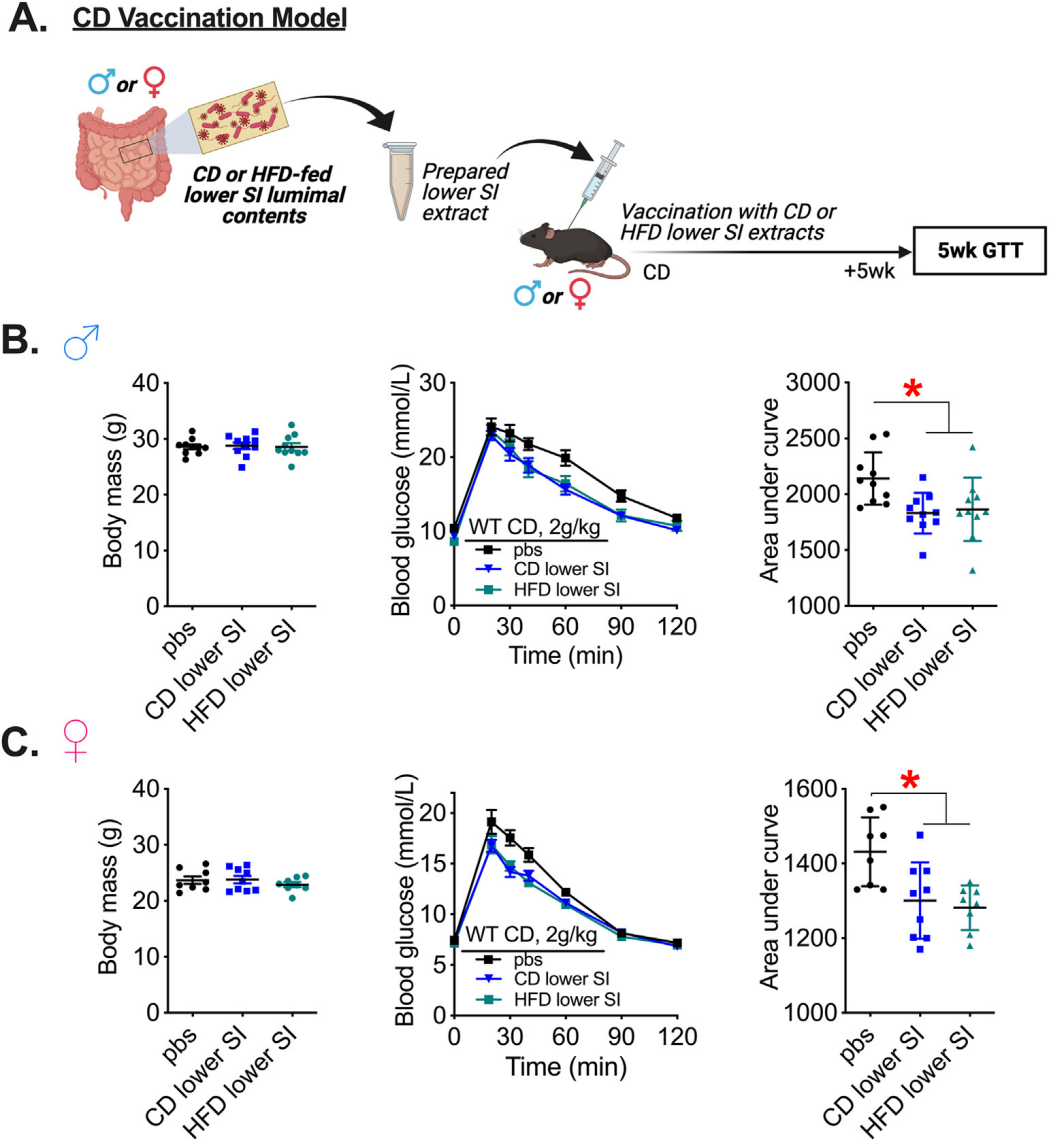
**Microbiota-based vaccination with bacterial extracts derived from lean or obese mice improves blood glucose control in lean mice independent of sex.** A) Experimental vaccination model of control diet (CD)-fed male or female mice given a single subcutaneous injection with an extract prepared from the lower small intestinal (SI) luminal contents of CD- or high fat diet (HFD)-fed male or female mice, respectively. Glucose tolerance was assessed in all vehicle-injected and lower SI-vaccinated mice 5 weeks after the vaccination event. B) Body mass, blood glucose vs. time and quantified area under the curve during a glucose tolerance test (GTT) (2 g/kg, i.p.) in CD-fed WT male mice 5 weeks after vaccination with CD lower SI extract or HFD lower SI extract (n = 10). C) Body mass, blood glucose vs. time and quantified area under the curve during a GTT (2 g/kg, i.p.) in CD-fed WT female mice 5 weeks after vaccination with CD lower SI extract or HFD lower SI extract (n = 8–9). ∗Denotes significant difference from control group injected with saline (pbs) determined by one-way ANOVA (p < 0.05). Each symbol represents a mouse and other values shown are the mean +/− SEM.

Diet is a major factor altering composition of the microbiota and even short-term changes in the relative amounts of ingested macronutrients produces rapid and distinct shifts in the microbial populations [49]. Since sex also alters the composition of the gut microbiota [50], we tested whether an interaction exists between diet and sex to influence glycemic control after a microbiota-based vaccination. We used lower SI extracts derived from male and female mice fed a CD or HFD and measured glycemia in mice vaccinated with lower SI contents from the same sex (Figure 3A). We found that lower SI extracts from male and female mice fed a CD or HFD lowered blood glucose during a GTT in lean male and female mice without changes in body mass (Figure 3B,C). Taken together, our results show that postbiotics derived from the lower SI of either lean or obese mice fed HFD possess similar blood glucose-lowering properties in both lean male and lean female mice.

### 3.3 Microbiota-based vaccination engages NOD2 to alter glycemic control

**Figure 4:**
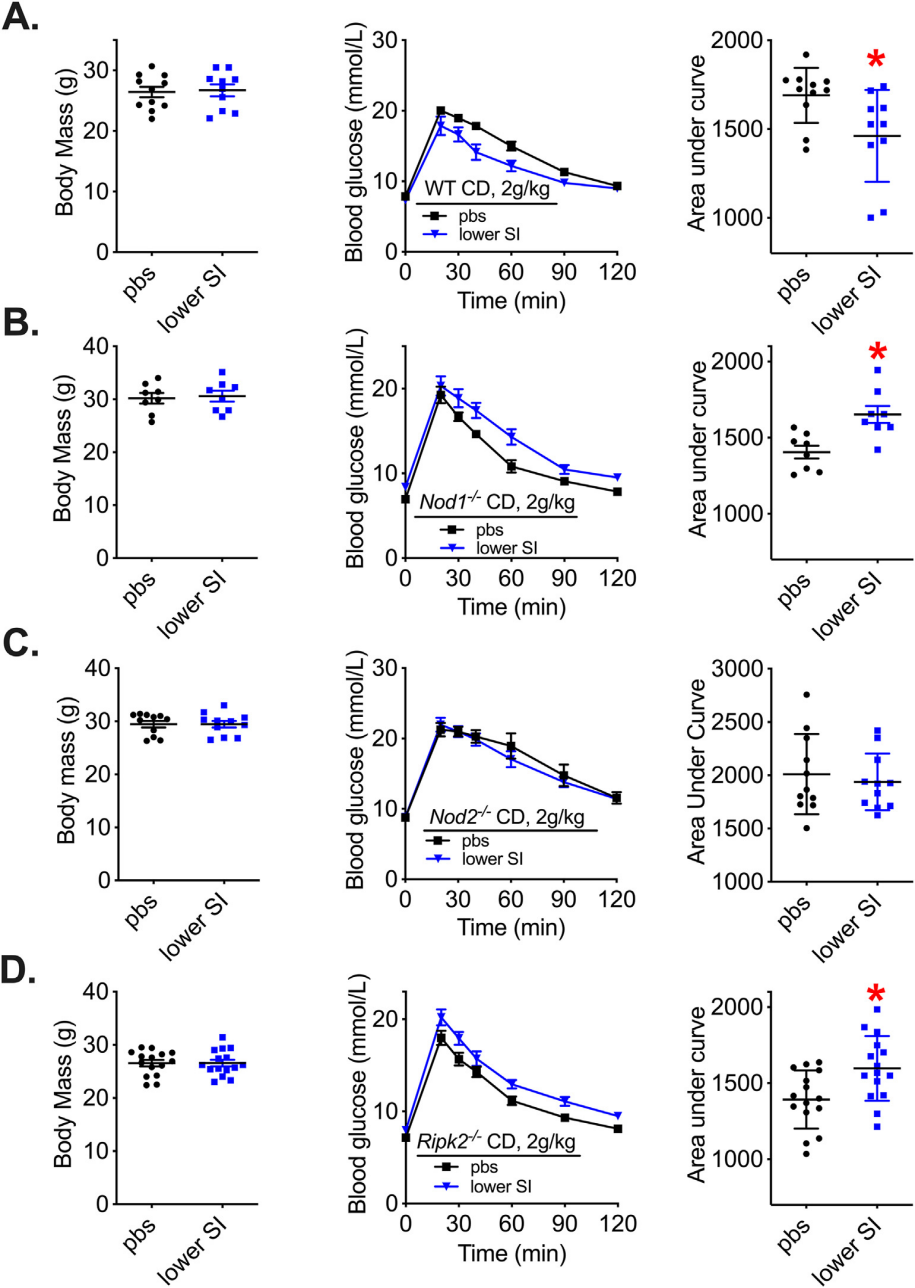
**Microbiota-based vaccination engages NOD-RIPK2 to alter glycemia in lean male mice.** A) Body mass, blood glucose vs. time and quantified area under the curve during a glucose tolerance test (GTT) (2 g/kg, i.p.) in control diet (CD)-fed wild type (WT) male mice 5 weeks after vaccination (n = 10–11). B) Body mass, blood glucose vs. time and quantified area under the curve during a GTT (2 g/kg, i.p.) in CD-fed Nod1^−/−^ male mice 5 weeks after vaccination (n = 8). C) Body mass, blood glucose vs. time and quantified area under the curve during a GTT (2 g/kg, i.p.) in CD-fed Nod2^−/−^ male mice 5 weeks after vaccination (n = 11). D) Body mass, blood glucose vs. time and quantified area under the curve during a GTT (2 g/kg, i.p.) in CD-fed Ripk2^−/−^ male mice 5 weeks after vaccination (n = 15). ∗Denotes significant difference from control group injected with saline (pbs) determined by t-test (p < 0.05). Each symbol represents a mouse and other values shown are the mean +/− SEM.

T-cell-mediated adaptive immunity is required for improved glucose control after a bacterial vaccination strategy in mice [43]. It was not known whether innate immune components are required. We tested whether the bacterial cell wall sensors NOD1 and NOD2 and a common adaptor Receptor-interacting serine/threonine-protein kinase 2 (RIPK2) propagate changes in glycemia after microbiota-based vaccination in mice. CD-fed wild-type (WT), *Nod1*^*−/−*^*, Nod2*^*−/−*^*,* and *Ripk2*^*−/−*^ male mice were vaccinated with lower SI bacterial extracts from CD-fed WT male mice. Our results confirm that WT mice have significantly lower blood glucose levels during a GTT five weeks after vaccination (Figure 4A). We found that *Nod1*^*−/−*^ mice had higher blood glucose during a GTT five weeks after vaccination (Figure 4B). We found that *Nod2*^*−/−*^ mice had no change in blood glucose during a GTT five weeks after vaccination (Figure 4C). *Ripk2*^*−/−*^ mice had higher blood glucose during a GTT five weeks after vaccination (Figure 4D), which paralleled the blood glucose response in *Nod1*^*−/−*^ mice after vaccination. Taken together, these results show that a peptidoglycan sensing NOD-RIPK2-mediated innate immune signalling pathway is required for a microbiota vaccination strategy to improve blood glucose control in male mice. We found NOD2 is required for bacterial vaccination strategy to alter blood glucose in mice, whereas deletion of *Nod1* or *Ripk2* worsens glycemic control when male mice are injected subcutaneously with bacterial extracts.

### 3.4 A prime-boost microbiota-based vaccination improves glucose control in obese male mice

**Figure 5:**
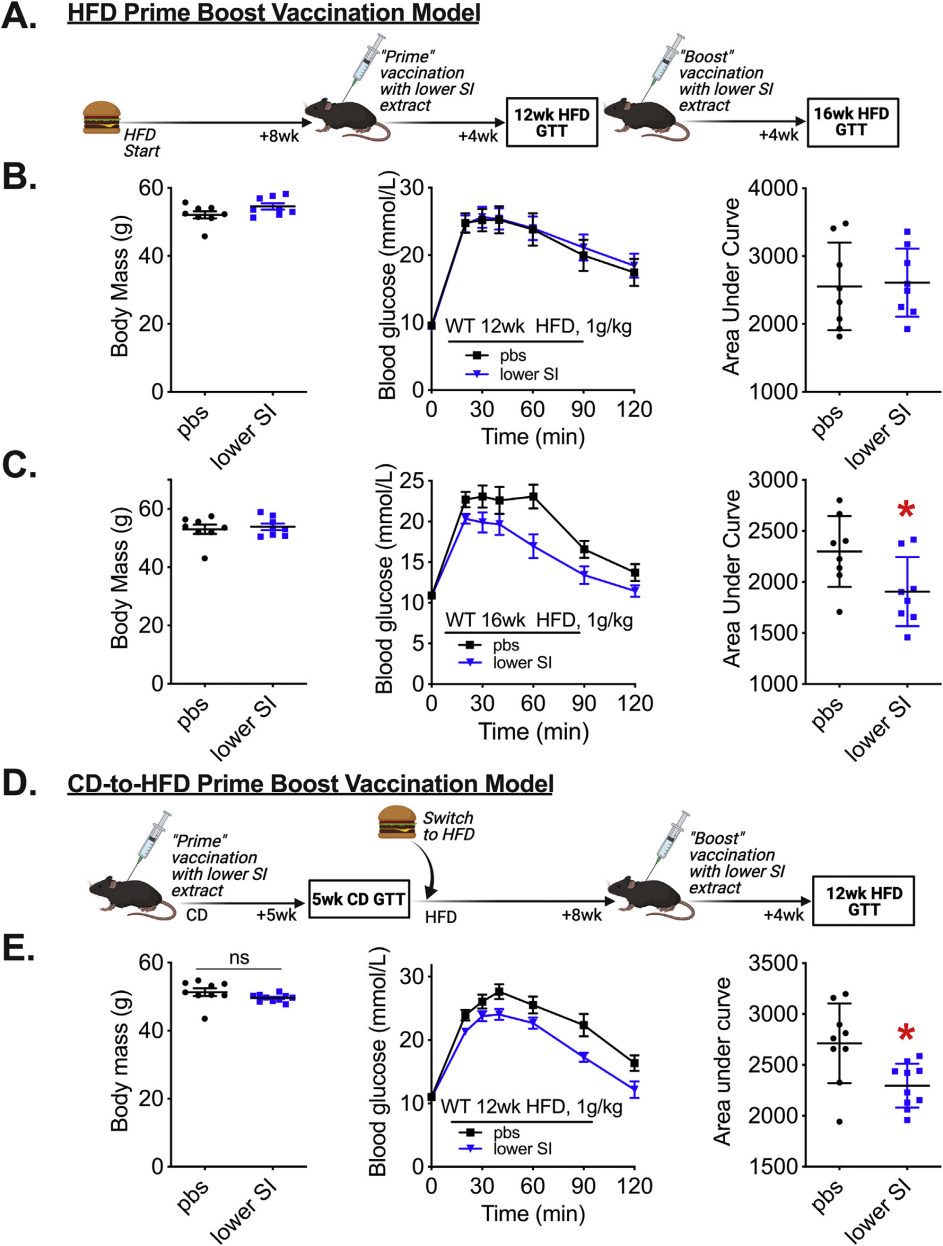
**A prime-boost vaccination strategy is required to improve glucose control in obese male mice.** A) Vaccination model during diet-induced obesity where mice were fed a HFD for 8 weeks before the first lower SI extract injection and glucose tolerance was assessed 4 weeks after the initial injection. A second ‘boost’ injection of lower SI extract was delivered, and glucose tolerance was assessed 4 weeks after the second injection, which equated to a total of 16 weeks of HFD feeding. B) Body mass, blood glucose vs. time and quantified area under the curve during a glucose tolerance test (GTT) (1 g/kg, i.p.) in 12wk HFD-fed WT male mice vaccinated a single time, according to Fig 5A (n = 8). C) Body mass, blood glucose vs. time and quantified area under the curve during a GTT (1 g/kg, i.p.) in 16wk HFD-fed WT male mice vaccinated twice, according to Fig 5A (n = 8). D) Experimental vaccination model of mice given a “prime” injection of lower SI extract during control diet (CD)-feeding in mice. Five weeks after the prime injection, mice were switched to a HFD for 8 weeks and then a second “boost” injection of lower SI extract was delivered. Four weeks later, after 12 total weeks of HFD feeding, glucose tolerance was assessed. E) Body mass, blood glucose vs. time and quantified area under the curve during a GTT (1 g/kg, i.p.) in 12wk HFD-fed WT male mice vaccinated twice according to Fig 5D (n = 9–10). ∗Denotes significant difference from control group injected with saline (pbs) determined by t-test (p < 0.05). Each symbol represents a mouse and other values shown are the mean +/− SEM.

We next sought to determine whether diet-induced obesity altered the efficacy of microbiota-based vaccination on glycemic control. Male mice were fed a HFD for 8 weeks and then injected with lower SI bacterial extracts (Figure 5A). This initial “prime” vaccination did not alter body mass or blood glucose control 4 weeks later when the mice had been on a HFD for a total of 12 weeks (Figure 5B). These mice were then given a second (boost) injection with lower SI extracts (Figure 5A). Four weeks after the boost vaccination, mice had lower blood glucose levels during a GTT, despite no change in body mass (Figure 5C). We then tested whether the initial prime vaccination could be given in lean CD fed mice, followed the second boost vaccination in the same mice after HFD-feeding (Figure 5D). This prime-boost vaccination spanning a change from CD to HFD feeding lowered blood glucose levels during a GTT without a change in body mass (Figure 5E). Taken together, these data show that a ‘prime-boost’ vaccination strategy with lower SI bacterial extracts is needed to improve blood glucose control during diet-induced obesity in male mice. A single injection of bacterial extracts was insufficient to improve blood glucose control in obese mice. Blood glucose levels could be lowered if the first (prime) and boost injection were given to mice before and after the onset of diet-induced obesity, respectively.

### 3.5 Muramyl dipeptide is not sufficient to improve blood glucose control when administered as a vaccination

**Figure 6:**
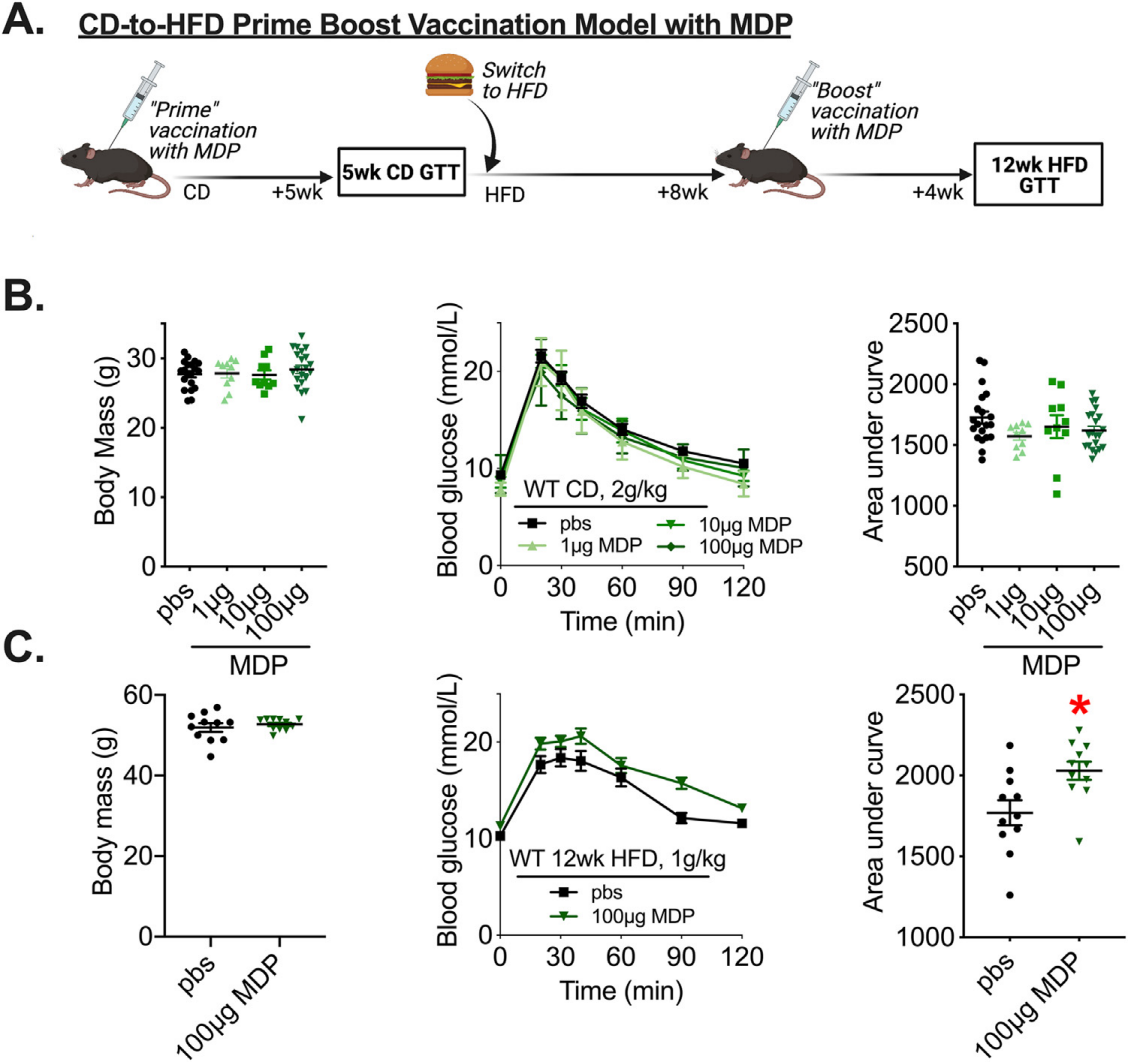
**MDP is not sufficient to improve blood glucose control using a vaccination-style delivery.** A) Vaccination model of CD-fed mice given a single injection of muramyl dipeptide (MDP). Glucose tolerance was assessed 5 weeks after the initial injection and mice were switched to a HFD for 8 weeks before a second injection of MDP was delivered. Four weeks after the second injection, after 12 total weeks of HFD feeding, glucose tolerance was assessed. B) Body mass, blood glucose vs. time and quantified area under the curve during a glucose tolerance teste (GTT) (2 g/kg, i.p.) in control diet (CD)-fed WT male mice injected once with 1 μg, 10 μg, or 100 μg MDP (n = 10–21). C) B) Body mass, blood glucose vs. time and quantified area under the curve during a GTT (1 g/kg, i.p.) in 12wk HFD-fed WT male mice injected twice (i.e., Prime and boost injections) with 100 μg MDP (n = 11). ∗Denotes significant difference from the control group injected with saline (pbs) determined by t-test (p < 0.05). Each symbol represents a mouse and other values shown are the mean +/− SEM.

We have previously shown that three consecutive injections of MDP (100 μg each) requires NOD2 to lower blood glucose levels during a GTT in obese male and female mice [36,37]. We found that *Nod2*^*−/−*^ mice were refractory to changes in blood glucose after a vaccine-like lower SI extract injection in mice (Figure 4C). We next tested single injection of different doses of MDP to determine if MDP was a component present in the lower SI extract that was sufficient to improve blood glucose control in our vaccination model (Figure 6A). Blood glucose levels were not altered five weeks after a single injection of MDP (1 μg, 10 μg and 100 μg doses) in CD-fed male mice (Figure 6B). A second injection of MDP after 8 weeks of HFD produced a significant increase in blood glucose levels during a GTT after mice were on a HFD for a total of 12 weeks (Figure 6C). These data indicate that MDP is not the pivotal extract ingredient responsible for improved blood glucose control caused by microbiota-based vaccination.

### 3.6 Microbiota-based vaccination increases bacterial extract-specific IgG in the ileum and alters the composition of the gut microbiome

**Figure 7:**
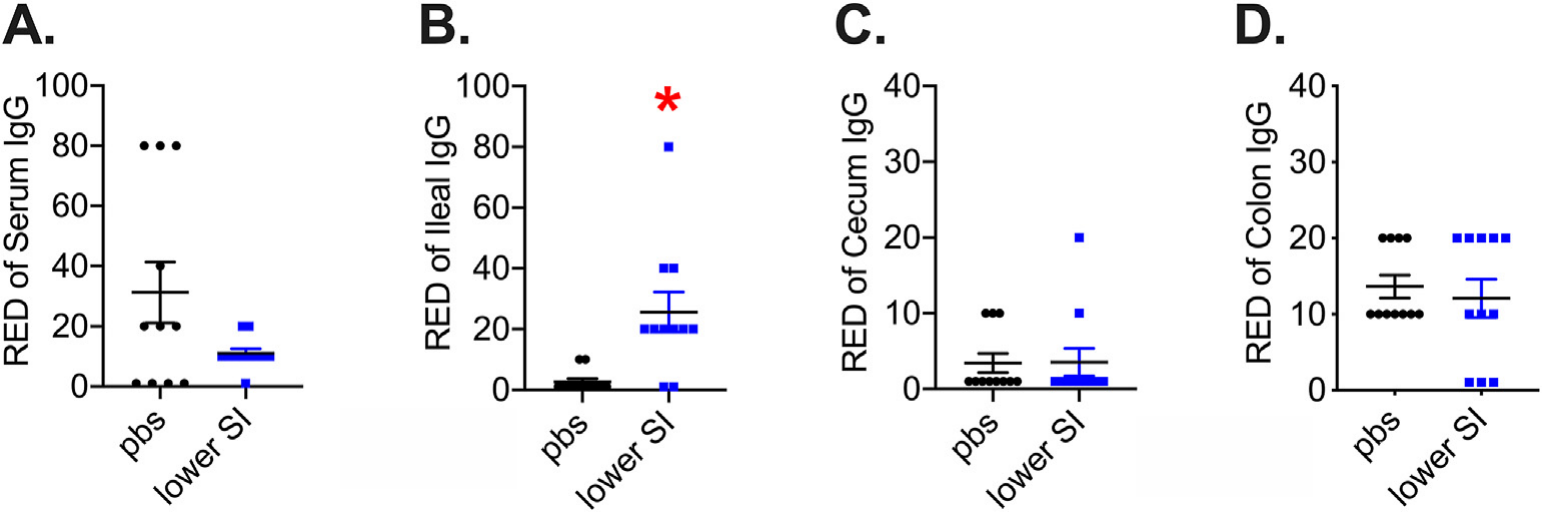
**Quantification of extract-specific systemic and intestinal IgG levels in vaccinated mice.** Reciprocal endpoint dilution (RED) of specific Immunoglobin-G (IgG) raised against lower small intestinal (SI) extract antigens in A) Serum, B) Ileum, C) Cecum, and D) Colon of male mice vaccinated with the same lower SI extracts (n = 11/group for all tissues). ∗Denotes significant difference from control group injected with saline (pbs) determined by t-test (p < 0.05). Each symbol represents a mouse and other values shown are the mean +/− SEM.

**Supplemental Figure 3:**
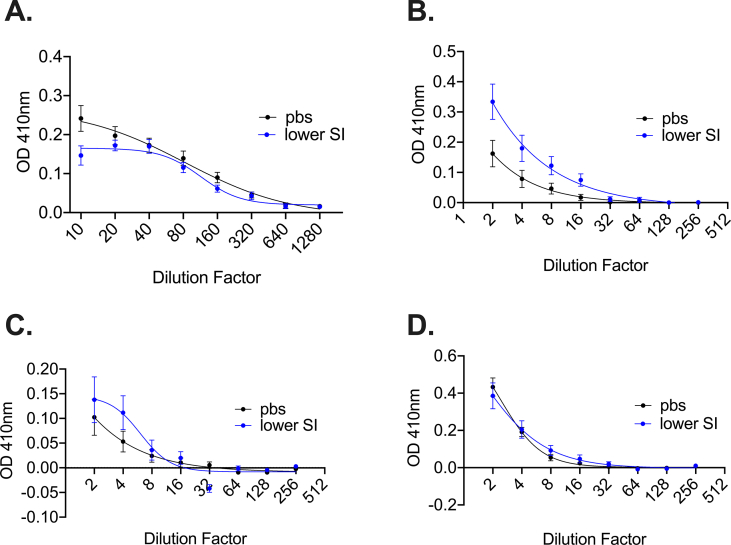
**Quantification of IgG levels directed against the extract antigens in vaccinated mice.** Serial dilutions of A) serum, B) ileum, C) cecum, and D) colon samples for detection of specific Immunoglobin-G (IgG) raised against lower small intestinal (SI) extract antigens in mice vaccinated with the same lower SI extracts (n = 11/group for all tissues). IgG was detected in mice 42 days after a single subcutaneous injection of lower SI extract (5000x dilution) or saline (pbs). Values shown are mean +/− SEM.

A previous study showed that subcutaneous injection of a proximal gut bacterial extract caused a modest increase in total circulating immunoglobin-G (IgG) in mice [43]. However, total IgG levels in the blood do not necessarily capture compartmentalized adaptive immune or immunoglobin responses raised against specific antigens. An immune response after microbiota-based vaccination with intestinal extracts could produce a robust increase in IgG directed against the components in the bacterial extract, despite little or no difference in total IgG concentration. We analysed the serum and different gut segments of vaccinated mice using ELISA to detect antibodies specific to components of the intestinal extract 42 days after vaccination of CD-fed mice. Our results show that serum levels of SI extract-specific IgG were unchanged in the serum of vaccinated mice (Figure 7A). We found an increase in the reciprocal endpoint dilution of IgG raised against the lower SI extract antigens in the lower SI, but not cecum or colon in mice after a microbiota-based vaccine (Figure 7B–D; see Supplemental Figure 3 for full serial dilution curves). These data suggest that subcutaneous injection with a lower SI bacterial extract elicits a specific B cell-mediated IgG response that is compartmentalized to the lower SI of vaccinated mice.

**Figure 8:**
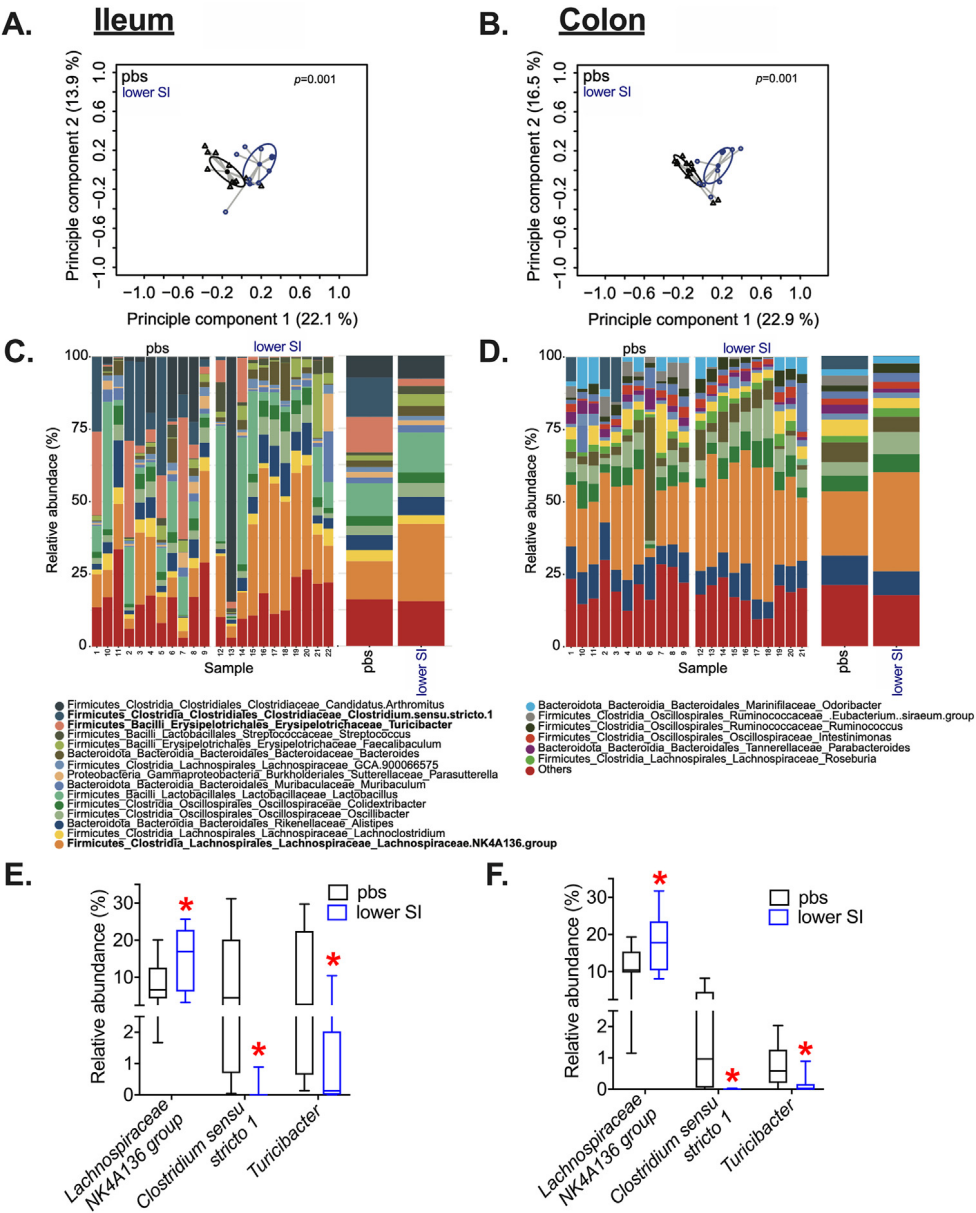
**Microbiota-based vaccination alters ileal and colon gut microbiota composition.** Principle coordinates analysis (PCoA) performed on Bray–Curtis distances in A) ileum and B) colon samples of CD-fed mice 42 days after vaccination with lower SI extract (blue circles) or pbs (black circles). Relative abundance of bacteria resolved to the phylum level in C) ileum and D) colon samples (with each bar representing an individual mouse) and the top three significant taxonomic differences in E) ileum and F) colon samples from CD-fed mice 42 days after vaccination with lower SI extract or pbs (n = 10–11, p < 0.05).

Based on these compartmentalized changes to intestinal immunity, we next sought to test if the composition of the microbial communities in the proximal or distal gut were altered by vaccination. Amplicon-based sequencing was used to obtain the taxonomic bacterial profile in ileum and colon 42 days after vaccination in CD-fed male mice. PCoA scatterplots on Bray–Curtis distance revealed a significant degree of dissimilarity between the bacterial communities found in the ileum and colon of vaccinated mice (Figure 8A–B). This vaccination-driven clustering of bacterial populations was associated with changes in taxa within the phylum Firmicutes (Figure 8C–D). *Lachnospiraceae.NK4a136.group* was increased after lower SI-vaccination, and this finding was consistent across the different gut niches analyzed (Figure 8E–F). After microbiota-based vaccination, the relative abundance of *Lachnospiraceae.NK4a136.group* increased to approximately 1/5th of the entire pool of sequences in both the lower SI and colon (Figure 8E–F). The relative abundance of *Clostridium.senso.strictu.1* and *Tenericutes* were decreased in the lower SI and in the colon after the microbiota-based vaccination (Figure 8E–F). These data suggest that gut bacteria, specifically *Lachnospiraceae.NK4a136.group,* are responsive to a microbiota-based vaccination and associated with increased IgG responses in the SI and long-lasting improvements in blood glucose control.

**Supplemental Figure 4:**
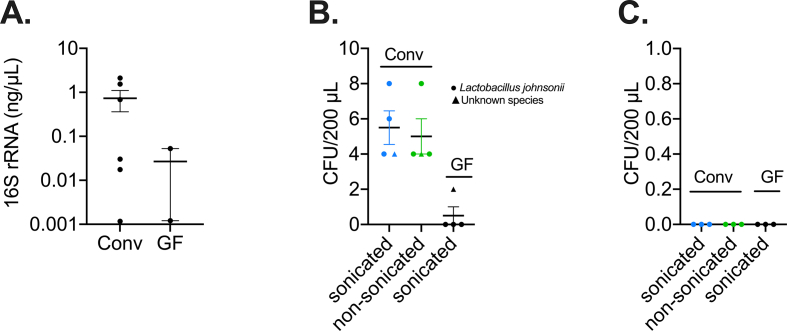
**Bacterial 16S quantification and bacterial viability in extracts.** A) Quantification of 16s rRNA concentration using broad range 16S qPCR in bacterial extracts derived from lower SI segments from conventionalized (Conv) mice harbouring a complex microbiota or germ-free (GF) mice (n = 2–6). B) Colony Forming Unit (CFU) counts from viable bacteria present in sonicated and non-sonicated lower SI extracts cultivated in BHI agar. C) CFU counts of viable bacteria obtained from sonicated or non-sonicated SI extracts that underwent spore germination with ethanol shock and were cultivated in BHI supplemented with rumen fluid. Identification of bacterial colonies was done using MALDI-TOF spectrometry.

It is difficult to establish the exact dose of each bacterial component contained in the microbiota-based extract prepared from each gut segment. We quantified 16S rRNA in the extracts prepared from the upper and lower SI in conventional (i.e., colonized) and germ-free mice (Supplemental Figure 4). We also showed that conventional bacterial extracts had a small number of live bacteria (∼5 CFU per injection volume), although this number was higher than extracts prepared from germ-free mice despite sonication (Supplemental Figure 4). We found no evidence of bacterial spores (Supplemental Figure 4). We identified that some of live bacteria in the extract were *Lactobacillus johnsonii*, which is consistent with the fact that lactobacilli possess cell wall envelopes that are difficult to break [51].

## 4 Discussion

Commensal bacteria engage innate and adaptive immunity, which can alter host metabolism. The intestine has many barriers that limit how bacteria influence host immunity and metabolism. There is emerging evidence that bacteria can subvert the intestine and that T2D dictates the type and amount of bacteria that can penetrate into metabolic tissues that control blood glucose [30]. Smaller molecules, such as bacterial cell wall components and metabolites, can also translocate across the intestinal barrier and engage immune response in host tissues that influence blood glucose control [52,53]. For example, microbiota-derived bacterial cell wall components such as meso-DAP-containing muropeptides and certain types of LPS promote inflammation, lipolysis, and insulin resistance [[52], [53], [54], [55], [56]]. Bacterial MDP promotes immune tolerance and insulin sensitivity [36,37]. How these opposing bacterial factors, particularly in a complex microbial extract that contains a plethora of bacterial metabolites and components, alter blood glucose control is unknown. One previous report showed that a single injection of a bacterial extract derived from the lower SI improved blood glucose control in mice, and this was linked to a systemic adaptive immune response [43]. Our results show that a microbiota-based vaccination generates a compartmentalized adaptive immune response where IgG directed against the antigens contained within the lower SI bacterial extract is subsequently increased in lower SI of mice receiving the injection. Our results show that improvements in blood glucose control after microbiota-based vaccination requires an innate immune NOD2-RIPK2-signalling pathway in the mice injected with bacterial extracts. Our results define the innate immune bacterial detection pathway and compartmentalized adaptive immune responses that are associated with improvements in blood glucose after injection of postbiotic bacterial components in a SI-derived bacterial extract.

Despite this need for NOD2 to propagate the glucose effects of a microbiota-based vaccination, a single subcutaneous injection of the well-known NOD2 ligand, MDP, was not sufficient to generate a robust long-lasting improvement in blood glucose control. It is possible that unclassified NOD2 ligands (beyond MDP) could be factors in bacterial extracts that improve blood glucose control. It is plausible that unknown bacterial factors within the bacterial extract engage innate immune receptors beyond NOD2, where the interaction of multiple innate immune pathways require NOD2 to drive an IgG-mediated adaptive immune response in the SI, and subsequently changes in the composition of the intestinal microbiome and glycemia. The identities of these other bacterial factors are not clear, but other bacterial components that engage separate innate immune responses could participate in an integrated response that alters blood glucose. For example, an injection of flagellin can improve blood glucose control in mice by engaging TLR5 and adaptive immune responses that can protect against aspects of inflammatory and metabolic diseases [57]. It is beyond the scope of the current paper to test all the possible bacterial factors that could interact to elicit long-lasting changes in blood glucose control. It is noteworthy that injection of a specific concentration of SI or cecal-derived bacterial extracts was required for improvements on blood glucose control, whereas extracts derived from the large intestine (or feces) did not alter blood glucose. Cecal extracts promoted lower blood glucose at lower concentrations (10,000x and 20,000x) compared to lower SI extracts (5000x). This could be related to the type of bacteria, type of bacterial components, or concentration of bacteria, which are much higher in the cecum and distal gut. Determining the concentration and gut-segment-dependent effects may help to elucidate the identity of postbiotic components that contribute to improved blood glucose control.

We have resolved that NOD2 is a critical component of innate immunity that mediates improved glycemic responses to microbiota-based immunization in male mice. We also found that deletion of NOD1 or RIPK2 worsened blood glucose control after microbiota-based vaccination. RIPK2 is the common adapter for both NOD1 and NOD2 and our data show that the NOD1-RIPK2 pathway protects against dysglycemia after subcutaneous injection of bacterial extracts. How or why this occurs is not known; it is possible that augmented TLR4 immune and lipid metabolism responses that can occur in the absence of RIPK2 could be mediators of dysglycemia [58]. We have consistently shown that repeated MDP engagement of NOD2 is an innate immune component that improves blood glucose control [36,37,59]. Based on our current data, we propose a new model where the small intestinal luminal contents contain a complex milieu of postbiotic components derived from the commensal microbiota that can either raise or lower glycemia and NOD1-RIPK2 versus NOD2-RIPK2 signalling dictates the immune response and subsequent net effects on glycemic control.

We found that changes in blood glucose and the compartmentalized intestinal IgG response were associated with changes in resident SI and colon microbial communities in vaccinated mice. The abundance of *Lachnospiraceae.NK4a136.group* was significantly increased in the ileum and colon of vaccinated mice. *Lachnospiraceae.NK4a136.group* is a potential butyrate-producer associated with metabolic fitness and gut homeostasis [60,61] and probiotic activity in mice [62]. We found that *Lachnospiraceae.NK4a136.group* constituted ∼20% of the sequenced bacterial taxa in vaccinated mice, which makes it a strong candidate for future testing related to glycemic control. Although *Lachnospiraceae.NK4a136.group* was increased in both the lower SI and colon of vaccinated mice, bacterial vaccination only increased IgG responses in the ileum. Our data do not directly link compartmentalized adaptive immunity to changes in the composition of the microbiota within each gut segment. It is plausible that microbial vaccination-induced increases in IgG in the lower SI influence the local gut microbiota in the same region, where compositional changes in the microbiome carry over into the distal gut (i.e., colon), but directionality in this host–microbe relationship has not yet been established. Although most of our experiments were focused on male mice, we did find similar improvements in glycemic control in female mice after a microbiota-based vaccine, suggesting a sex-independent effect on glycemia. However, a more thorough analysis of any sex-specific responses to microbiota-based vaccination across the life-course is warranted, given the sex- and age-related differences in gut microbiota composition, immunity, and metabolism [50].

## 5 Conclusions

In summary, we found that a simple microbiota-based vaccination procedure using subcutaneous injection of an SI- (or cecum)-derived bacterial extract elicits a localized IgG response specifically directed against the antigens in the bacterial extract and promotes improvements in blood glucose control in male and female mice. The SI-derived extracts required bacteria and engaged the NOD2-RIPK2 signalling pathway to improve blood glucose control in male mice. Obese male mice had to receive prime and boost injections for improvements in blood glucose control. There is much interest in manipulating the gut microbiota to improve metabolism during obesity. Given that some probiotic and FMT strategies aimed at combating metabolic disease have limitations that rely on live bacteria establishing a transient niche in the gut, investigation of postbiotics that promote long-term changes in blood glucose is warranted. Our results show that the net effect of injecting a specific concentration of postbiotics derived from the SI or cecum produces a long-lasting improvement in blood glucose control in mice.

## Author contributions

BDM researched data and wrote the manuscript. AKT, NGB, FFA, GP, JGW, HDS researched data. MSM, MGS, DMS contributed to design and discussion. JDS derived the hypothesis, wrote the manuscript and is the guarantor of the study.

## Data and code availability

All data and custom R scripts are available on request to the corresponding author.

## Financial support

10.13039/501100000024Canadian Institutes of Health Research (FDN-154295)
